# Knowledge, perception, and satisfaction of postpartum women about newborn hearing screening in two private Brazilian maternity hospitals

**DOI:** 10.1590/2317-1782/e20230326en

**Published:** 2025-02-24

**Authors:** Khalil Fouad Hanna, Aline Tocchini Pascoinelli Cremonesi, Maria Regina Torloni, Giovanna Dalo Ferreira

**Affiliations:** 1 Departamento de Triagem Auditiva Neonatal, Grupo Santa Joana - São Paulo (SP), Brasil.; 2 Núcleo de Apoio a Pesquisas e Publicações, Grupo Santa Joana - São Paulo (SP), Brasil.

**Keywords:** Neonatal Screening, Hearing Loss, Hearing, Postpartum Period, Patient Satisfaction, Consumer Health Information, Health Knowledge, Attitudes and Practice, Knowledge

## Abstract

**Purpose:**

To assess the knowledge, perception, and satisfaction of postpartum women about newborn hearing screening and investigate factors associated with lack of knowledge about the test.

**Methods:**

Cross-sectional analytical study conducted in two private Brazilian maternity hospitals. Participants were postpartum women with newborn infants eligible for hearing screening. After the hearing test, they answered an anonymous written questionnaire to assess their knowledge about, perception of, and satisfaction with the test. The characteristics of participants with and without knowledge about the test were compared using the Chi-square test. Variables with P <0.20 were included in the logistic regression.

**Results:**

The study included 470 postpartum women (74.1% had a university degree). Nearly 42% (n=195) had no prior knowledge about the test. Among those with prior knowledge, the main sources of information were having a previous child who had undergone the test (50.5%), and family/friends (26.2%). Primiparity (aOR 5.01, 95% CI 3.27-7.69), lack of information about the test during antenatal care (aOR 3.67, 95% CI 2.01-6.70), and no family member with hearing loss (aOR 2.00, 95% CI 1.16-3.47) were variables associated with the lack of knowledge about the test. Almost all participants (98.7%) perceived the test as very important, and 94.3% were totally satisfied with it.

**Conclusion:**

Even though newborn hearing screening became mandatory in Brazil over a decade ago, a large proportion of postpartum women in two private hospitals had no prior knowledge about the test. However, the vast majority perceive the test as very important and are highly satisfied with it.

## INTRODUCTION

Congenital hearing loss (HL), defined as hearing impairment present at birth, occurs in approximately 1.7 per 1,000 live births. This incidence is up to ten times higher in neonates with risk factors for hearing loss^(1-3)^. Universal newborn hearing screening (NHS) aims to facilitate the early identification of infants with HL, regardless of the presence of risk factors, thereby enabling referral for diagnostic assessments and appropriate interventions^(2,4)^. Early detection of HL is crucial, as diagnosis and the initiation of interventions within the first months of life have been shown to correlate strongly with improved outcomes in auditory function, as well as language, speech, and cognitive development^(2,4,5)^.

Current estimates indicate that NHS is accessible to only around one third of the global population, with its availability primarily concentrated in high-income countries and regions^(6)^. In 2010, a federal law mandated that all children born in Brazilian hospitals and maternity wards had to undergo NHS free of charge^(7)^. In 2018, the average NHS coverage in Brazil was 67.6%, although large variations between different regions were observed^(8)^. While NHS coverage exceeds 95% in the southern and southeastern regions of Brazil, certain states in the northeastern region report coverage rates of less than 25%^(9)^.

Lack of knowledge, along with parents´ negative perceptions and dissatisfaction with hearing screening tests, are factors that may contribute to the discontinuation of follow-up care for infants with suspected HL^(10-12)^. Several international studies have explored users' knowledge and perceptions regarding NHS^(13-17)^. However, most of the Brazilian studies on this topic were conducted prior to the 2010 legislation that made NHS mandatory, included relatively small sample sizes, or did not concurrently assess parents' knowledge, perception, and satisfaction with NHS^(18-23)^. This study is warranted given the relevance of the issue and the absence of recent national research about NHS.

The main objective of this study was to evaluate the knowledge of contemporary puerperal women about NHS, as well as their perception of and satisfaction with the test. The secondary objective was to identify factors associated with participants' lack of knowledge about NHS.

## METHODS

The study was approved by the Institution´s Research Ethics committee (Approval No. 4.379.545) and all participants provided written informed consent.

This analytical cross-sectional study was conducted between January and August 2021 by professionals from the hearing screening sector at two private maternity hospitals in the city of São Paulo. All patients at these two hospitals are either covered by health insurance or pay directly for services (out-of-pocket). The study employed non-probabilistic convenience sampling, with the investigators enrolling the first 235 women who met the selection criteria and consented to participate in the study at each of the two hospitals.

The study included women who were representative of the general population giving birth in private Brazilian maternity hospitals. Participants were women aged > 20 years, with rooming-in newborns eligible for NHS. Exclusion criteria included women who were not fluent in Portuguese, those receiving psychotropic drugs, and women with intellectual, mental, auditory, visual or physical impairments that could interfere with the comprehension or completion of written questionnaires. Participants were subsequently divided into two groups based on their prior knowledge about NHS.

The study employed an anonymous written questionnaire (without participant identification) which was developed by the investigators based on similar studies^(13-19,23,24)^. The initial version of the questionnaire was pilot-tested with a group of five puerperal volunteers, leading to adjustments in the wording of certain questions for improved clarity. The final version of the questionnaire had two sections (Appendix 1). The first section consisted of seven questions designed to collect sociodemographic and obstetric information. The second section included seven questions aimed at assessing participants´ knowledge about NHS, sources of information, the importance attributed to the test, and their satisfaction with their baby having undergone the test. All questions were closed-ended, employing dichotomous (yes/no) or multiple-choice responses, or utilized a Likert scale (Appendix 1). The first question inquired whether the participant had any prior knowledge or information about NHS (henceforth referred to as “NHS knowledge”) with a Yes/No response. Based on this response, participants were categorized into two distinct groups (those with and those without NHS knowledge), which were subsequently compared in the statistical analyses.

Speech therapists responsible for NHS at the participating hospitals collected all data. Prior to conducting the test, speech therapists routinely provide an explanation to mothers regarding the procedure, as well as the purpose of the screening test. After the test, they deliver the results to the mothers. During the study period, the speech therapist introduced herself to each woman, explained the objectives and methods of the study before conducting the newborn screening test, and invited all eligible women to participate. Those who accepted the invitation signed an informed consent form. Following the completion of the NHS test and the provision of results, each participant was given an anonymous written questionnaire to complete individually. After distributing the questionnaire, the speech therapist left the room and returned 5-10 minutes later to collect the completed forms. If the patient had any questions regarding the questionnaire, the speech therapist provided clear, objective responses in a neutral tone to avoid influencing the participant´s answers. If the questionnaire was not completed within the allotted time, the participant was given an additional 10 minutes to finish. Once completed, the questionnaire was placed in a brown envelope, which was sealed in the presence of the participant and subsequently stored in a folder alongside the envelopes of other participants.

Participants' responses were transferred to Excel© spreadsheets (version 2016). The characteristics of the participants and their answers to the questionnaire are presented as absolute frequencies, percentages, and measures of central tendency and dispersion. A univariate analysis was initially conducted to assess the relationship between maternal characteristics (independent variables) and lack of knowledge about NHS (dependent variable). Variables with a p-value < 0.20 in the univariate analysis (Chi-square test) were included in the multivariate logistic regression analysis (Wald methods) to examine the independent effects of these variables on “lack of NHS knowledge”. In the multivariate logistic regression analysis, only variables with a p-value <0.05 were retained in the final model. Crude (OR) and adjusted (aOR) odds ratios, alongside their respective 95% confidence intervals (CI), are reported for each independent variable. The Hosmer-Lemeshow test was used to determine the goodness of fit of the logistic regression model. All analyses were conducted using STATA17 software (StataCorp LP, College Station, United States).

## RESULTS

Participants´ age ranged from 20 to 47 years (mean: 32.3, standard deviation: 5.0). The majority were White, married, had a university degree, were employed, had no family members with hearing loss, and were primiparas (first time mothers) (Table 1).

**Table 1:** Main characteristics of 470 study participants, São Paulo, 2021

Characteristic	n (%)
Age, years	
20-29	125 (26.6)
30-39	318 (67.7)
> 40	27 (5.7)
Race/color	
White	316 (67.2)
Mixed	112 (23.8)
Black	36 (7.7)
Yellow	6 (1.3)
Marital status	
Married	310 (66.0)
Single	74 (15.7)
Common-law marriage	72 (15.3)
Divorced or separated	14 (3.0)
Education	
< 12 years	10 (2.1)
12 years	66 (14.0)
Incomplete higher education	46 (9.8)
Higher education or more	348 (74.1)
Employment	
Yes	372 (79.2)
No	20 (4.2)
No information	78 (16.6)
Family member(s) with hearing loss	
Yes	85 (18.1)
No	385 (81.9)
Parity*	
1	269 (57.2)
> 2	201 (42.8)

* Parity: number of previous deliveries, including the current one

Approximately 42% (n=195) of participants reported having no prior knowledge about the newborn hearing screening test. Among the 275 participants with some level of NHS knowledge, the primary sources of information were prior experience with another baby who had undergone the test (50.5%), family or friends (26.2%), and the internet or social media (25.1%). Over 80% of participants indicated that their obstetrician had never mentioned NHS during prenatal care. Nearly 99% of participants regarded the test as very important, approximately 90% were totally satisfied with the information provided by the speech therapists prior to the test, and over 91% had the opportunity to ask questions about the test. Over 94% were totally satisfied with the screening test performed on their baby (Table 2).

**Table 2:** Knowledge, perception and satisfaction of 470 puerperal women about newborn hearing screening, São Paulo, 2021

Variable	n (%)
Already knew or had heard about NHS	
Yes	275 (58.5)
No	195 (41.5)
Source of knowledge about NHS (n=275)*	
Another child had done NHS	139 (50.5)
Family or friends	72 (26.2)
Internet, social media	69 (25.1)
TV, radio	5 (1.8)
Magazines, newspapers	3 (1.1)
Other sources	16 (5.8)
Obstetrician mentioned NHS during prenatal care	
Yes	85 (18.1)
No	385 (81.9)
Importance attributed to NHS**	
6 - 8	3 (0.6)
9	3 (0.6)
10	464 (98.7)
Satisfaction with information received from speech therapist about the test and its results	
totally dissatisfied	1 (0.2)
partially dissatisfied	1 (0.2)
neither satisfied nor dissatisfied	2 (0.4)
partially satisfied	45 (9.6)
totally satisfied	421 (89.6)
Had the chance to ask the speech therapist all the questions that she wanted about the test	
Yes	428 (91.1)
No	42 (8.9)
General satisfaction with the test***	
1- 5	1 (0.2)
6 - 8	16 (3.4)
9	10 (2.1)
10	443 (94.3)

* More than one source could be reported

** Likert scale: 1=not important at all, 10=extremely important

*** Likert scale: 1=totally dissatisfied, 10=totally satisfied

**Caption:** NHS = Newborn Hearing Screening, TV = Television

In the univariate analysis (Table 3), four variables (age, parity, family history of hearing loss, and information about the test given by the obstetrician during prenatal care) were associated (p < 0.20) with lack of knowledge about NHS and were selected for inclusion in the multivariate logistic regression analysis. The remaining variables analyzed were not associated with lack of NHS knowledge.

**Table 3:** Variables associated with lack of knowledge about the newborn hearing screening test: univariate analysis. São Paulo, 2021

Variables	Prior knowledge		P*
	Yes (N=275)	No (N=195)	
Age, years			
<35	172 (62.5)	135 (69.2)	0.1609**
> 35	103 (37.5)	60 (30.8)	
Race/color			
White or Yellow	186 (67.6)	136 (69.7)	0.5110
Mixed	66 (24.0)	46 (23.6)	
Black	23 (8.4)	13 (6.7)	
Marital status			
Married or common-law marriage	226 (82.2)	156 (80.0)	0.6331
Other	49 (17.8)	39 (20.0)	
Higher education			
Yes	201 (73.1)	147 (75.4)	0.6512
No	74 (26.9)	48 (24.6)	
Employment			
Yes	217 (78.9)	155 (79.5)	0.5395
No	14 (5.1)	6 (3.1)	
No information	44 (16.0)	34 (17.4)	
Parity***			
1	117 (42.5)	152 (77.9)	<0.0001**
> 2	158 (57.5)	43 (22.1)	
Family member(s) with hearing loss			
Yes	59 (21.5)	26 (13.3)	0.033**
No	216 (78.5)	169 (86.7)	
Obstetrician mentioned NHS during prenatal care			
Yes	68 (24.7)	17 (8.7)	<0.0001**
No	207 (75.3)	178 (91.3)	

* Chi square test

** Variables with P < 0.20 selected for inclusion in multivariate logistic regression

*** Parity: number of previous deliveries, including the current one

**Caption:** NHS = Newborn hearing screening

In the final multivariate regression model (Table 4), three of the four variables with p < 0.20 in the univariate analysis were found to be significantly associated with lack of NHS knowledge (p < 0.05). Primiparas (first-time mothers) were 5.01 times more likely to lack knowledge about NHS compared to multiparas (women with prior childbirth experience). Participants who had not received information about the test during prenatal care were 3.67 times more likely to have no knowledge about NHS compared to those who had received such information. Participants who had no family members with hearing loss were 2.00 times more likely to lack knowledge about NHS than those with family members who had hearing loss. According to the Hosmer-Lemeshow test, the model was adequate to explain the factors associated with lack of NHS knowledge (p = 0.7911

**Table 4:** Variables associated with lack of knowledge about the newborn hearing screening test: multivariate analysis. São Paulo, 2021

Variables	OR	(95% CI)	Adjusted OR	(95% CI)	P*
Primiparity**	4.77	(3.15-7.23)	5.01	(3.27-7.69)	<0.0001
Obstetrician did not mention NHS during prenatal care	3.44	(1.95-6.07)	3.67	(2.01-6.70)	<0.0001
Absence of family member with hearing loss	1.78	(1.07-2.94)	2.00	(1.16-3.47)	0.013

* Wald test

** Primiparity: women with only one delivery

**Caption:** OR = Odds ratio; CI = Confidence Interval; NHS = Newborn Hearing Screening

## DISCUSSION

Despite their high level of education, four out of ten women who had just given birth in two private Brazilian maternity hospitals reported no prior knowledge about NHS. Factors associated with lack of knowledge about NHS included primiparity, not receiving information about the screening test during prenatal care, and the absence of family members with hearing loss. However, the vast majority of study participants regarded NHS as a very important test and were totally satisfied with the test performed on their baby.

Consistent with our findings, studies in various countries report that 27% to 80% of pregnant and postpartum women have insufficient or no knowledge about NHS^(13,16)^. Brazilian studies conducted after NHS became mandatory report that 42% to 81% of participants have no knowledge about the test^(20-23)^. These data suggest that NHS is often conducted without parents fully understanding what is being done, or the benefits of early detection of HL. According to the framework proposed by Sekhon et al, patients´ lack of understanding about an intervention and how it works (“coherence”) is one of the factors influencing the acceptability of health interventions, including screening tests^(25)^. Additionally, lack of knowledge about NHS is associated with increased parental anxiety regarding the test and lower levels of satisfaction with the procedure^(15,23,26,27)^.

Parents' knowledge, perception, and attitude towards newborn screening tests are influenced by the information they receive. The timing, format, source, and amount of information about the test are critical factors for parents´ understanding about NHS. In this study, the primary source of information about NHS was prior experience with the test for another child, while fewer than 20% of participants reported receiving any information from their obstetrician about the test during prenatal care. In other studies, the main sources of information about NHS included online reading materials, family and friends, educational activities during prenatal care, and mass media communication channels^(16,18,19)^. The literature suggests that parents prefer to receive information about NHS in the form of pamphlets during pregnancy, rather than at the time of hospital admission or in the immediate postpartum period^(15,28)^.

The perceived importance of the test and the high level of satisfaction with NHS reported by our participants are also frequently, although not unanimously, reported in the existing literature^(13-17,23,26)^. Parental satisfaction with the test is particularly important because individuals who are satisfied with NHS are more likely to be collaborative and attend follow-up visits for their child^(12,24)^.

This study has several implications for practice. The findings highlight the need to provide more information about NHS to Brazilian women during pregnancy, particularly for those who are having their first child. This responsibility could be addressed by prenatal care providers and could involve offering concise, objective information in various formats (oral communication, written pamphlets, audio materials, digital content)^(28)^. Evidence suggests that delivering information about NHS during the third trimester of pregnancy significantly enhances parents' satisfaction with the test and may improve the effectiveness of screening and early treatment programs for children with congenital HL^(29)^.

This study raises several questions that warrant further investigation. To gain a more comprehensive understanding about the knowledge, attitude, and satisfaction of our population regarding NHS, similar studies should be conducted in other public and private hospitals across Brazil. Future studies should employ probabilistic sampling methods and include women with diverse socioeconomic and obstetric profiles, as well as mothers of babies in intensive care units. To improve the methodological rigor of future studies, it would be important to translate and validate tools such as the questionnaire developed by Mazlan et al. in 2006^(24)^, or the more recent instrument proposed by Graham et al.^(30)^ to assess mothers' knowledge and attitudes toward NHS.

The main strength of this study is its status as the largest Brazilian study about puerperal women's knowledge, perception, and satisfaction with NHS. The two main study limitations were the use of convenience sampling and the adoption of a questionnaire developed by the authors. While Australian researchers developed a questionnaire in 2006 to assess parents' satisfaction with NHS^(24)^, it has not yet been translated or validated into Brazilian Portuguese. Another limitation, common to studies relying on self-reported data, is the potential for memory bias, which may have influenced participants´ responses regarding prior knowledge about NHS and its source. Finally, the findings of this study are not generalizable to populations with different characteristics or to participants managed in public hospitals.

## CONCLUSION

More than a decade after the implementation of mandatory NHS in Brazil, four out of ten women giving birth in private maternity hospitals have no prior knowledge about the test, despite the fact that the majority of these women have higher education. Nevertheless, most women perceive the test as being very important, and nearly all express strong satisfaction with it. Predictive factors for lack of knowledge about NHS include primiparity, not receiving information about the test during prenatal care, and the absence of family members with hearing loss. These findings underscore the need to provide more information about the test during prenatal care, particularly for women who are pregnant for the first time.

## Appendix 1 Knowledge, perception and satisfaction about newborn hearing screening

Date: ___/___/202___ Hospital:____________________________________

1. What is your age? _____years

2. You consider yourself:

()White

()Mixed race

()Black

()Yellow

()Another race/color

3. What is your marital status?

()Married

()Single

()Common-law marriage

()Divorced/Separated

()Widow

4. What is your degree of instruction?

() incomplete primary education (less than 9 years)

() complete primary education (9 years)

() incomplete secondary education (10-11 years)

() complete secondary education (12 years)

() incomplete higher education (enrolled in college/university but did not graduate)

() complete higher education (college/university graduate)

() complete or incomplete post-graduation

5. Do you have a paid job or employment? ()No, ()Yes

6. Does anyone in your family have hearing loss? ()No, ()Yes

7. How many times did you give birth (including your current delivery):_____

8. Prior to today, did you have any knowledge about or had heard of the Newborn Hearing Screening test, also known as the “Baby Hearing Test”? ()No, ()Yes

8.a. If you answered Yes, where did your knowledge about the Newborn Hearing Screening test come from (you can select more than one option):

() this test was done in another child that I had

() family or friends

() internet, social media

() TV, radio

() magazines, newspapers

() other sources: describe ____________________________________________

9. During your prenatal care appointments, did your obstetrician say anything about the Newborn Hearing Screening test? ()No, ()Yes

10. On a scale of 1 to 10, how important do you think the Newborn Hearing Screening test is for your baby. 1 means that the test has no importance at all and 10 means that the test is extremely important .

No importance Extremely

at all important

**Table:**

1	2	3	4	5	6	7	8	9	10

11. How satisfied are you with the information that you received from the speech therapist about the Newborn Hearing Screening test and its results?

() totally dissatisfied

() partially dissatisfied

() neither satisfied nor dissatisfied

() partially satisfied

() totally satisfied

12. Did you have the chance to ask the speech therapist all the questions that you wanted about the Newborn Hearing Screening test?

() No, () Yes

13. On a scale of 1 to 10, how satisfied are you in general with the Newborn Hearing Screening test that was just done on your baby? 1 means that you are totally dissatisfied with the test and 10 means that you are totally satisfied with the test.

Totally Totally

dissatisfied satisfied

**Table:**

1	2	3	4	5	6	7	8	9	10
