# Supplementary material for: Knowledge, perception, and satisfaction of postpartum women about newborn hearing screening in two private Brazilian maternity hospitals
Source: Codas. 2025 Feb 24;37(2):e20230326. doi: 10.1590/2317-1782/e20230326en (PMC11864762; doi:10.1590/2317-1782/e20230326en)
Supplement: Translation

# Translation: Conhecimento, percepção e satisfação de puérperas sobre o exame de triagem auditiva neonatal em duas maternidades privadas brasileiras

**DOI:** 10.1590/2317-1782/e20230326pt

**Authors:** Khalil Fouad Hanna, Aline Tocchini Pascoinelli Cremonesi, Maria Regina Torloni, Giovanna Dalo Ferreira

**Affiliations:** 1 Departamento de Triagem Auditiva Neonatal, Grupo Santa Joana - São Paulo (SP), Brasil.; 2 Núcleo de Apoio a Pesquisas e Publicações, Grupo Santa Joana - São Paulo (SP), Brasil.

**Keywords:** Triagem Neonatal, Perda Auditiva, Audição, Período Pós-Parto, Satisfação do Paciente, Informação de Saúde ao Consumidor, Conhecimentos, Atitudes e Prática em Saúde, Conhecimento

## Abstract

**Objetivo:**

Avaliar o conhecimento, percepção e satisfação de puérperas em relação à triagem auditiva neonatal e investigar fatores associados ao desconhecimento do exame.

**Método:**

Estudo transversal analítico realizado em duas maternidades privadas brasileiras. As participantes eram puérperas com recém-nascidos elegíveis para o exame. Após a realização da triagem auditiva, elas responderam a um questionário impresso anônimo com perguntas sobre seu conhecimento, percepção e satisfação com o exame. As características das participantes com e sem conhecimento do exame foram comparadas usando o teste do Qui-quadrado. As variáveis com P <0.20 foram incluídas no modelo de regressão multivariada.

**Resultados:**

O estudo incluiu 470 puérperas (74,1% com nível superior completo). Cerca de 42% (n=195) não tinham nenhum conhecimento prévio sobre o exame. Entre as que tinham algum conhecimento prévio, as principais fontes de informação eram a realização do mesmo exame em outro filho (50,5%) e familiares/amigos (26,2%). Ser primípara (ORa 5,01, IC 95% 3,27-7,69), não ter recebido informações sobre o exame durante o pré-natal (ORa 3,67, IC 95% 2,01-6,70) e não ter familiar com perda auditiva (ORa 2,00, IC 95% 1,16-3,47) foram variáveis associadas ao desconhecimento do exame. Quase todas participantes (98,7%) atribuíram grande importância ao exame e 94,3% estavam totalmente satisfeitas com o mesmo.

**Conclusão:**

Apesar da triagem auditiva neonatal ter se tornado obrigatória no Brasil há mais de uma década, uma grande proporção das puérperas atendidas em duas maternidades privadas desconhecia o exame. Porém quase todas percebem o exame como muito importante e relatam alto grau de satisfação com sua realização.

## INTRODUÇÃO

A deficiência auditiva (DA) congênita, definida como perda auditiva presente no nascimento, afeta cerca de 1,7 em cada 1000 nascidos vivos, sendo até dez vezes maior nos bebês com fatores de risco para perda auditiva^(1-3)^. A triagem auditiva neonatal (TAN) universal visa identificar precocemente os recém nascidos com DA, com ou sem indicadores de risco, para encaminhá-los para exames diagnósticos definitivos e subsequente tratamento^(2,4)^. A detecção precoce da DA é fundamental pois o diagnóstico e a implementação de intervenções nos primeiros meses de vida estão diretamente associados a melhores resultados no desenvolvimento da função auditiva, da linguagem, da fala e do processo de aprendizagem^(2,4,5)^.

Na atualidade, estima-se que a TAN está disponível para apenas cerca de um terço da população mundial, concentrando-se predominantemente nos países e regiões de alta renda^(6)^. Em 2010, uma lei federal tornou obrigatória a realização gratuita da TAN em todas as crianças nascidas em hospitais e maternidades do Brasil^(7)^. Em 2018, a cobertura média nacional de TAN era de 67,6%, com grande variabilidade regional^(8)^. Enquanto a TAN tem cobertura superior a 95% nas regiões sul e sudeste, alguns estados do nordeste têm cobertura inferior a 25%^(9)^.

A falta de conhecimento, assim como percepções negativas e a insatisfação dos pais com a triagem auditiva, são fatores que podem contribuir para a perda de seguimento dos recém-nascidos (RNs) com suspeita de DA^(10-12)^. Consequentemente, diversos estudos internacionais avaliaram o conhecimento e a percepção dos usuários sobre a TAN^(13-17)^. A maioria dos estudos nacionais sobre esse tema foi realizada antes da lei de 2010 que tornou obrigatória a realização do exame no Brasil, incluiu relativamente poucos participantes, ou não avaliou simultaneamente o conhecimento, a percepção e satisfação dos pais em relação à TAN^(18-23)^. A relevância do tema e a falta de estudos recentes na literatura nacional justificam a realização deste estudo.

O objetivo principal deste estudo foi avaliar o conhecimento, a percepção, e a satisfação de puérperas contemporâneas em relação à triagem auditiva neonatal. O objetivo secundário foi investigar fatores associados ao desconhecimento das participantes a respeito da TAN.

## MÉTODO

O estudo foi aprovado pelo comitê de ética em pesquisa institucional (n° 4.379.545) e todos os participantes assinaram um termo de consentimento livre e esclarecido.

Este estudo transversal analítico foi realizado por profissionais do setor de triagem auditiva de duas maternidades do setor privado na cidade de São Paulo entre janeiro e agosto de 2021. Estas maternidades atendem exclusivamente pacientes cujos gastos são cobertos por planos de saúde ou recursos próprios. O estudo envolveu uma amostragem não probabilística, de conveniência. Em cada um dos hospitais, as investigadoras incluíram no estudo as primeiras 235 mulheres que preencheram os critérios de seleção e que concordaram em participar do estudo.

O estudo avaliou mulheres representativas da população geral que dão à luz em maternidades brasileiras privadas. Incluímos mulheres com > 20 anos com RNs que estavam em alojamento conjunto e que eram elegíveis para realização da TAN. Mulheres com qualquer das seguintes características foram consideradas inelegíveis: sem domínio da língua portuguesa, em uso de drogas psicotrópicas, com déficit intelectual, mental, auditivo, visual ou físico que pudesse dificultar a compreensão ou o preenchimento de questionários escritos. As participantes foram divididas em dois grupos, conforme seu conhecimento prévio quanto ao exame de triagem auditiva neonatal.

O estudo envolveu a aplicação de um questionário impresso anônimo (sem identificação das participantes) desenvolvido pelos investigadores, baseado em estudos semelhantes^(13-19,23,24)^. A primeira versão do questionário foi testada em um grupo piloto de cinco puérperas voluntárias e o enunciado de algumas perguntas foi ajustado para maior clareza. A versão final do questionário tinha duas partes (Anexo 1). A primeira parte tinha sete perguntas sobre dados sociodemográficos e obstétricos; a segunda parte tinha sete perguntas para avaliar o conhecimento e fonte de informação sobre a TAN, a percepção da importância do exame e a satisfação das participantes com a TAN. Todas as perguntas eram fechadas, com respostas dicotômicas (sim/não), de múltipla escolha ou em uma escala Likert (Anexo 1). A primeira pergunta indagava se a participante tinha algum conhecimento ou informação prévia sobre a TAN (doravante designado como “conhecimento”) com resposta fechada (Sim/Não). A resposta a esta pergunta foi usada para classificar as respondentes em dois grupos independentes que foram comparados nas análises estatísticas (com versus sem conhecimento sobre TAN).

Fonoaudiólogas responsáveis pela TAN nos hospitais participantes fizeram a coleta dos dados. Antes da realização da TAN, todas as fonoaudiólogas que atuam nesses hospitais explicam rotineiramente o que será feito e qual é a finalidade do exame; após o exame, as fonoaudiólogas explicam o resultado do mesmo. Durante o período do estudo, depois de se apresentar a cada paciente e antes de realizar a TAN, a fonoaudióloga explicou os objetivos e a metodologia da pesquisa e convidou todas as mulheres elegíveis a participarem do estudo. As que aceitaram participar assinaram o termo de consentimento informado. Após a realização da TAN e a informação sobre o resultado, cada participante recebeu um questionário impresso anônimo que deveria ser preenchido de forma individual. A fonoaudióloga saiu do quarto após a entrega do questionário, retornando cerca de 5-10 minutos depois. Caso a paciente tivesse dúvidas em relação a qualquer pergunta, a fonoaudióloga fornecia esclarecimentos objetivos em tom neutro, para não influenciar as respostas. Caso o questionário não estivesse preenchido dentro do prazo estipulado, era concedido um tempo adicional de mais 10 minutos. O questionário preenchido foi colocado em um envelope pardo que foi lacrado na frente da participante e colocado em uma pasta com os envelopes das outras participantes.

As respostas das participantes foram transcritas para planilhas do software Excel© (versão 2016). Apresentamos as principais características das participantes e as respostas ao questionário com números absolutos, porcentagens, medidas de tendência central e de dispersão. Inicialmente, realizamos uma análise univariada para avaliar a relação entre as características maternas (variáveis independentes) e o desconhecimento sobre a TAN (variável dependente). As variáveis com P<0,20 na análise univariada (teste do Qui-quadrado) foram incluídas na análise de regressão logística multivariada (métodos de Wald) para investigar o efeito independente dessas variáveis sobre o “desconhecimento da TAN”. Na análise de regressão logística multivariada, apenas as variáveis com valor de P<0,05 foram incluídas no modelo final. As razões de chances bruta (OR) e ajustada (ORa) e seus respectivos intervalos de confiança (IC) de 95% são apresentados para cada variável independente. O ajuste do modelo de regressão logística foi avaliado pelo teste de Hosmer-Lemeshow. Todas as análises foram realizadas no programa STATA17 (StataCorp LP, College Station, Estados Unidos).

## RESULTADOS

A idade das participantes variou de 20 a 47 anos (média: 32,3, desvio padrão: 5,0), a maioria era branca, casada, tinha ensino superior completo, exercia trabalho remunerado, não tinha familiares com perda auditiva e tinha acabado de dar à luz pela primeira vez (primíparas) (Tabela 1).

**Tabela 1:** Principais características das 470 participantes do estudo, São Paulo, 2021

Característica	n (%)
Idade, anos	
20-29	125 (26,6)
30-39	318 (67,7)
> 40	27 (5,7)
Raça/cor	
Branca	316 (67,2)
Parda	112 (23,8)
Negra	36 (7,7)
Amarela	6 (1,3)
Estado Civil	
Casada	310 (66,0)
Solteira	74 (15,7)
União estável	72 (15,3)
Divorciada ou separada	14 (3,0)
Escolaridade	
< 12 anos	10 (2,1)
12 anos	66 (14,0)
superior incompleto	46 (9,8)
superior completo ou mais	348 (74,1)
Trabalho remunerado	
Sim	372 (79,2)
Não	20 (4,2)
Não respondeu	78 (16,6)
Algum familiar com perda auditiva	
Sim	85 (18,1)
Não	385 (81,9)
Paridade*	
1	269 (57,2)
> 2	201 (42,8)

* Paridade: número de partos anteriores, incluindo o parto atual

Cerca de 42% (n=195) das participantes declararam não ter nenhum conhecimento prévio sobre o exame de triagem auditiva neonatal. Entre as 275 que relataram ter algum conhecimento, as principais fontes de informação eram a experiência de ter realizado o exame em outro bebê (50,5%), familiares/amigos (26,2%), ou internet/redes sociais (25,1%). Mais de 80% das participantes declararam que seu obstetra não havia falado sobre a TAN durante o pré-natal. Cerca de 99% das participantes consideravam o exame extremamente importante, cerca de 90% estavam totalmente satisfeitas com as informações recebidas das fonoaudiólogas sobre o exame antes de sua realização e mais de 91% tiveram a oportunidade de fazer perguntas sobre o exame. Mais de 94% estavam totalmente satisfeitas com o exame realizado (Tabela 2).

**Tabela 2:** Conhecimento prévio, percepção e satisfação de 470 puérperas em relação ao exame de triagem auditiva neonatal, São Paulo, 2021

Variável	n (%)
Já conhecia ou já tinha ouvido falar do exame de TAN	
Sim	275 (58,5)
Não	195 (41,5)
Fonte de informação sobre a TAN (n=275)*	
Fez TAN em outro filho	139 (50,5)
Família ou amigos	72 (26,2)
Internet, redes sociais	69 (25,1)
TV, rádio	5 (1,8)
Revistas, jornais	3 (1,1)
Outros	16 (5,8)
Obstetra falou sobre a TAN durante o pré-natal	
Sim	85 (18,1)
Não	385 (81,9)
Grau de importância atribuído ao exame de TAN**	
6 - 8	3 (0,6)
9	3 (0,6)
10	464 (98,7)
Satisfação com as informações recebidas da fonoaudióloga sobre o exame e seu resultado	
totalmente insatisfeita	1 (0,2)
parcialmente insatisfeita	1 (0,2)
neutra	2 (0,4)
parcialmente satisfeita	45 (9,6)
totalmente satisfeita	421 (89,6)
Teve oportunidade de fazer todas as perguntas que desejava à fonoaudióloga sobre o exame	
Sim	428 (91,1)
Não	42 (8,9)
Grau de satisfação geral com o exame realizado***	
1- 5	1 (0,2)
6 - 8	16 (3,4)
9	10 (2,1)
10	443 (94,3)

* Mais de uma fonte poderia ser indicada

** Escala Likert: 1=nenhuma importância, 10=extremamente importante

*** Escala Likert: 1=totalmente insatisfeita, 10=totalmente satisfeita

**Legenda:** TAN = Triagem auditiva neonatal, TV = Televisão

Na análise univariada (Tabela 3), quatro variáveis (faixa etária, paridade, história de familiar com perda auditiva, e informação sobre o exame dada pelo obstetra durante o pré-natal) foram associadas (P < 0,20) ao desconhecimento sobre a TAN e selecionadas para inclusão na regressão logística multivariada. As outras variáveis analisadas não tiveram associação com o desconhecimento da TAN.

**Tabela 3:** Variáveis associadas ao desconhecimento do exame de triagem auditiva neonatal: análise univariada. São Paulo, 2021

Variáveis	Conhecimento prévio		P*
	Sim (N=275)	Não (N=195)	
Idade, anos			
<35	172 (62,5)	135 (69,2)	0,1609**
> 35	103 (37,5)	60 (30,8)	
Cor/Raça			
Branca ou Amarela	186 (67,6)	136 (69,7)	0,5110
Parda	66 (24,0)	46 (23,6)	
Negra	23 (8,4)	13 (6,7)	
Estado civil			
Casada ou união estável	226 (82,2)	156 (80,0)	0,6331
Outro	49 (17,8)	39 (20,0)	
Ensino superior completo			
Sim	201 (73,1)	147 (75,4)	0,6512
Não	74 (26,9)	48 (24,6)	
Trabalho remunerado			
Sim	217 (78,9)	155 (79,5)	0,5395
Não	14 (5,1)	6 (3,1)	
Sem informação	44 (16,0)	34 (17,4)	
Paridade***			
1	117 (42,5)	152 (77,9)	<0,0001**
> 2	158 (57,5)	43 (22,1)	
Familiar com perda auditiva			
Sim	59 (21,5)	26 (13,3)	0,033**
Não	216 (78,5)	169 (86,7)	
Obstetra falou sobre TAN no pré-natal			
Sim	68 (24,7)	17 (8,7)	<0,0001**
Não	207 (75,3)	178 (91,3)	

* Teste do Qui quadrado.

** As variáveis com valor de P < 0.20 foram selecionadas para inclusão na análise de regressão logística multivariada

*** Paridade: número de partos anteriores, incluindo o parto atual

**Legenda:** TAN: Triagem auditiva neonatal

No modelo final de regressão multivariada (Tabela 4), dentre as quatro variáveis com P< 0.20 na análise univariada, apenas três foram associadas ao desconhecimento da TAN (P < 0.05). As primíparas (mulheres que estavam dando à luz pela primeira vez) tiveram 5,01 vezes mais probabilidade de desconhecer a TAN do que as multíparas (mulheres que já haviam dado à luz antes), as participantes que não receberam informações sobre o exame durante o pré-natal tiveram probabilidade 3,67 maior de desconhecer a TAN do que as que receberam essa informação, e as participantes sem familiares com perda auditiva tiveram 2.00 vezes mais probabilidade de desconhecer a TAN do que aquelas com familiares com perda auditiva. Segundo o teste de Hosmer-Lemeshow, o modelo foi adequado para explicar os fatores associados ao desconhecimento prévio sobre a TAN (P=0,7911).

**Tabela 4:** Variáveis associadas ao desconhecimento do exame de triagem auditiva neonatal: análise multivariada, modelo final. São Paulo, 2021

Variáveis	OR Bruto	(IC 95%)	OR Ajustado	(IC 95%)	P*
Primiparidade**	4,77	(3,15-7,23)	5,01	(3,27-7,69)	<0,0001
Obstetra não falou sobre TAN no pré-natal	3,44	(1,95-6,07)	3,67	(2,01-6,70)	<0,0001
Ausência de familiar com perda auditiva	1,78	(1,07-2,94)	2,00	(1,16,3,47)	0,013

* P de Wald

** Primiparidade: mulheres com apenas um parto

**Legenda:** OR = Odds ratio, IC = Intervalo de confiança; TAN = Triagem auditiva neonatal

## DISCUSSÃO

Apesar de sua alta escolaridade, quatro em cada dez mulheres que haviam acabado de dar à luz em duas maternidades privadas brasileiras não tinham conhecimento prévio sobre a TAN. A primiparidade, não ter recebido informações sobre o exame durante o pré-natal e a ausência de familiares com deficiência auditiva foram fatores associados ao desconhecimento da TAN. Porém, a grande maioria das participantes do estudo percebia a TAN como um exame muito importante e estava totalmente satisfeita com a realização do mesmo.

Em concordância com nossos achados, 27%-80% das gestantes e puérperas entrevistadas em diversos países desconhecem ou têm conhecimento insuficiente sobre a TAN^(13,16)^ e os estudos brasileiros realizados depois da obrigatoriedade da TAN relatam que 42%-81% das participantes desconhecem o exame^(20-23)^. Esses dados indicam que, muitas vezes, a TAN é realizada sem que os responsáveis saibam do que se trata ou dos benefícios decorrentes do diagnóstico precoce da DA. Segundo a teoria proposta por Sekhon e colaboradores, a falta de compreensão do paciente sobre uma intervenção e como ela funciona (“coerência”) é um dos fatores que afeta a aceitabilidade de intervenções em saúde, incluindo exames de rastreamento^(25)^. A falta de conhecimento sobre a TAN também está associada à maior ansiedade parental com o exame e menor satisfação com o mesmo^(15,23,26,27)^.

O conhecimento, a percepção e a atitude dos pais em relação a exames de rastreamento neonatais são influenciadas pelas informações que eles recebem. O momento, o formato, a fonte e a quantidade de informação sobre o exame são importantes para que os pais entendam a TAN. Neste estudo, a experiência de ter realizado o exame em outro filho foi a principal fonte de informação sobre a TAN e menos de 20% das participantes relataram ter recebido informações do seu obstetra sobre o exame durante o pré-natal. Em outros estudos, as principais fontes de informação sobre TAN foram a leitura de material disponível online, amigos e familiares, ações educativas durante o pré-natal e os meios de comunicação em massa^(16,18,19)^. A literatura indica que os pais preferem receber panfletos sobre a TAN durante a gestação e não no momento da internação ou no pós-parto imediato^(15,28)^.

A percepção da importância do exame e o alto grau de satisfação com a TAN observadas entre as participantes deste estudo também são achados frequentes, porém não unânimes, na literatura^(13-17,23,26)^. A satisfação dos responsáveis com o exame é importante pois pais satisfeitos com a TAN são mais colaborativos e têm maior probabilidade de comparecer aos retornos programados para o seguimento da criança^(12,24)^.

Este estudo traz várias implicações para a prática. Os resultados apontam para a necessidade de oferecer mais informações sobre a TAN para as gestantes brasileiras, especialmente para as que não têm filhos anteriores. Esse papel poderia ser assumido pelos profissionais responsáveis pela realização do pré-natal e incluir a oferta de informações curtas e objetivas, em diversos formatos (oral, folhetos, áudio, digital)^(28)^. Existe evidência que a oferta de informações sobre a TAN no terceiro trimestre da gestação aumenta significativamente a satisfação dos pais com o exame e pode otimizar programas de rastreamento e tratamento precoce de crianças com DA congênita^(29)^.

Este estudo levanta diversas questões que devem ser respondidas em futuras pesquisas. Para uma visão mais abrangente sobre o conhecimento, a atitude e a satisfação da nossa população em relação à TAN, é necessário conduzir estudos semelhantes em outros hospitais brasileiros públicos e privados. Futuros estudos devem usar amostragens probabilísticas e incluir mulheres com outras características socioeconômicas e obstétricas, assim como mães de crianças internadas em unidade de terapia intensiva. Para aumentar a qualidade metodológica de futuras pesquisas semelhantes, seria importante traduzir e validar o instrumento desenvolvido por Mazlan e colaboradores em 2006^(24)^, ou o recente questionário proposto por Graham e colaboradores^(30)^ para avaliar o conhecimento e a atitude das mães sobre TAN.

O principal ponto forte deste estudo é o fato de ser a maior pesquisa brasileira sobre conhecimento, percepção e satisfação de puérperas sobre a TAN. As duas principais limitações do estudo foram o uso de uma amostra de conveniência e a aplicação de um questionário criado pelos próprios autores. Em 2006, pesquisadores australianos desenvolveram um questionário para avaliar a satisfação dos pais com a TAN^(24)^, porém, não existe versão traduzida e validada desse questionário para o Português do Brasil. Outra limitação deste estudo, comum a todos os estudos que envolvem dados auto-relatados, é a possibilidade de que o viés de memória tenha afetado as respostas referentes ao conhecimento prévio sobre TAN e a fonte dessa informação. Finalmente, os achados deste estudo não podem ser extrapolados para outras populações com diferentes características ou para participantes atendidos em hospitais públicos.

## CONCLUSÃO

Mais de uma década após a TAN ter se tornado obrigatória no Brasil, quatro em cada dez mulheres que deram à luz em maternidades privadas relatam desconhecer o exame, apesar da maioria ter nível superior. Porém, a maioria atribui grande importância ao exame e quase todas sentem-se muito satisfeitas com a realização do mesmo. A primiparidade, não ter recebido informações sobre o exame durante o pré-natal e a ausência de familiares com deficiência auditiva foram fatores preditivos para o desconhecimento da TAN. Estes resultados apontam para a necessidade de aumentar a oferta de informações sobre o exame durante o pré-natal, especialmente para as mulheres que estão grávidas pela primeira vez.

## Anexo 1. Questionário Conhecimento, percepção e satisfação com a Triagem Auditiva Neonatal

Data: ___/___/202___ Hospital:____________________________________

1. Qual é sua idade? _____anos

2. A Sra. se considera:

()Branca

()Parda

()Negra

()Amarela

()Outra raça/cor

3. Qual é seu Estado Civil?

()Casada

()Solteira

()União estável,

()Divorciada/Separada

()Viúva

4. Qual é sua Escolaridade?

() ensino fundamental incompleto (menos de 9 anos)

() ensino fundamental completo (9 anos)

() ensino médio incompleto (10-11 anos)

() ensino médio completo (12 anos)

() ensino superior incompleto (entrou na faculdade mas não terminou)

() ensino superior completo (concluiu uma faculdade)

() pós-graduação completa ou incompleta

5. A Sra. exerce algum trabalho remunerado? ()Não, ()Sim

6. A Sra. tem algum familiar com perda auditiva? ()Não, ()Sim

7. Quantas vezes a Sra. já deu à luz (contando com o parto atual):_____

8. Antes de hoje, a Sra. já conhecia ou já tinha ouvido falar do exame de Triagem Auditiva Neonatal, também conhecido por “Teste da orelhinha”? ()Não, ()Sim

8.a. Se respondeu Sim, como ficou sabendo sobre a Triagem Auditiva Neonatal (pode assinalar mais de uma opção):

() já fiz esse exame em outro filho(a)

() família ou amigos

() internet, redes sociais

() TV, radio

() revistas, jornais

() Outros: descreva ____________________________________________

9. Durante o pré-natal, seu médico obstetra falou sobre a Triagem Auditiva Neonatal? ()Não, ()Sim

10. Em uma escala de 1 até 10, assinale o grau de importância que a Sra. dá ao exame Triagem Auditiva Neonatal na vida do seu bebê. A nota 1 significa que o exame não tem nenhuma importância e a nota 10 significa que o exame é extremamente importante.

Nenhuma Extremamente

importância importante

**Table:**

1	2	3	4	5	6	7	8	9	10

11. Qual é seu grau de satisfação com as informações recebidas da fonoaudióloga sobre o exame de Triagem Auditiva Neonatal e seu resultado?

() totalmente insatisfeita

() parcialmente insatisfeita

() neutra

() parcialmente satisfeita

() totalmente satisfeita

12. A Sra. teve a oportunidade de fazer todas as perguntas que desejava à fonoaudióloga sobre o exame de Triagem Auditiva Neonatal?

() Não, () Sim

13. Em uma escala de 1 até 10, assinale seu grau de satisfação geral com o exame de Triagem Auditiva Neonatal que acabou de ser feito no seu bebê. A nota 1 significa que a Sra. está totalmente insatisfeita e a nota 10 significa que a Sra. está totalmente satisfeita.

Totalmente Totalmente

insatisfeita satisfeita

**Table:**

1	2	3	4	5	6	7	8	9	10
